# The Book World of Medicine and Science

**Published:** 1906-05-05

**Authors:** 


### The Book World of Medicine and Science.

City of London Directory, 1906. (Messrs. W. H. and L. Collingridge, 148-149 Aldersgate Street, London, E.C. Price 12s. 6d.)

The thirty-sixth annual issue of this Directory contains valuable information in regard to the City of London, the Corporation, livery companies, and other public bodies. The volume also records all the recent Government changes brought about by the new administration, an invaluable feature, inasmuch as most other works of reference went to press before the appointments were notified. These particulars, an excellent map, and a very complete list of individuals and firms doing business in the "one square mile" renders this book an indispensable work of reference to business men everywhere.

